# Red Cell Distribution Width is Associated with Future Incidence of Abdominal Aortic Aneurysm in a Population-Based Cohort Study

**DOI:** 10.1038/s41598-020-64331-7

**Published:** 2020-04-29

**Authors:** Jun Xiao, Yan Borné, Anders Gottsäter, Jingxue Pan, Stefan Acosta, Gunnar Engström

**Affiliations:** 1 Department of Cardiovascular Surgery, Fujian Medical University Union Hospital, Fuzhou, China; 2 Department of Clinical Sciences in Malmö, Lund University, Malmö, Sweden; 3 Vascular Centre, Department of Cardiothoracic and Vascular Surgery, Skåne University Hospital, Malmö, Sweden

**Keywords:** Predictive markers, Vascular diseases

## Abstract

Red cell distribution width (RDW) has been suggested to have a predictive potential for several cardiovascular diseases, but its association with abdominal aortic aneurysm (AAA) is unknown. We examined whether RDW is associated with the risk of AAA among 27,260 individuals from the population-based Malmö Diet and Cancer Study cohort. Data of baseline characteristics were collected during 1991–1996. Cox regression was used to estimate hazard ratios (HR) with 95% confidence intervals (CI) for AAA across quartiles of RDW. During a median follow-up of 21.7 years, 491 subjects developed AAA. After adjustment for other confounding factors, participants in the highest quartile of RDW experienced 61% increased risk of AAA as compared to those with the lowest quartile (HR = 1.61, CI = 1.20, 2.12). RDW showed similar relationship with severe (i.e. ruptured or surgically repaired) AAA or non-severe AAA (adjusted HR 1.58 and 1.60, respectively). The observed association between RDW and AAA risk was significant in current smokers (adjusted HR = 1.68, CI = 1.18, 2.38) but not in former smokers (adjusted HR = 1.13, CI = 0.72, 1.79), or never-smokers (adjusted HR = 1.77, CI = 0.74, 4.22). Elevated RDW is associated with increased future incidence of AAA, however the causal and pathophysiological mechanisms remain to be explored.

## Introduction

Red cell distribution width (RDW) is an easy, inexpensive, and rapid measurement of the heterogeneity of erythrocyte volumes. This measure is routinely calculated for most patients in whom a complete blood count is requested. It was originally developed for classification of anemias in clinical practice, in combination with mean corpuscular volume (MCV). For example, iron deficiency anemia is normally characterized by high RDW and low MCV, whereas vitamin B12 or folic acid deficiency is linked to high RDW and high MCV^1–3^.

Besides its traditional role in anemia diagnosis, an increasing body of studies documented relationships between high level of RDW and increased risk of cardiovascular diseases (CVDs), including coronary heart disease (CHD), ischemic cerebrovascular disease and peripheral artery disease, heart failure, and atrial fibrillation^4,5^.

Abdominal aortic aneurysm (AAA) is one of the most common CVDs with estimated prevalence rates between 1.3% and 8.9% in elderly men and between 1.0% and 2.2% in women^6^. AAA is a local arterial dilatation of the abdominal aorta, the pathogenesis of which differs from occlusive diseases although both entities are regarded as atherosclerosis-related diseases. AAA is often overlooked at early stages, since it is typically asymptomatic before rupture, when it becomes life-threatening^7–9^. However, until now, no effective drug medication is proposed to prevent formation and development of AAA^7,10^. So it is critical to distinguish individuals at high risk of AAA and provide them regular surveillance.

Studies of the relation between RDW and incidence of AAA is still lacking. Since previous studies have associated high RDW values with an increased risk of different cardiovascular diseases, we examined the hypothesis that RDW is related to an increased incidence of AAA. This study was performed in the population-based Malmö Diet and Cancer study.

## Results

### Incidence of AAA

During a mean follow-up of 21.7 years, a total of 491 subjects, of whom 371 were men and 120 were women, developed AAA, generating an incidence rate of 0.91 per 1,000 person-years in the whole population, 1.89 per 1,000 person-years in men, and 0.36 per 1,000 person-years in women. Mean age of 491 individuals who developed AAA was 61.0 ± 6.7 years at the baseline examination and 75.4 ± 6.7 years at the time of AAA diagnosis.

### Baseline characteristics

The distribution of baseline characteristics across quartiles of RDW is shown in Table 1. Age, ApoA1, white blood cell count, MCV, the proportion of female, daily smokers, and history of CHD increased with increasing RDW (all *p* < 0.001), whereas it was opposite for waist circumference, diastolic blood pressure, ApoB, ApoB/ApoA1 ratio, hemoglobin, the proportion of diabetes, and history of anti-hypertensive medication (all *p* < 0.001).

**Table 1:** Baseline characteristics across quartiles of red cell distribution width.

MDCS (n = 27260)	Quartile 1 (n = 6877)	Quartile 2 (n = 6946)	Quartile 3 (n = 6847)	Quartile 4 (n = 6590)	P
**RDW range**	28.1–38.4	38.5–40.4	40.5–42.7	42.8–72.9	
**Age(years)**	56.9 ± 6.9	58.0 ± 7.5	58.6 ± 7.9	59.2 ± 8	<0.001
**Sex(n,%)**					<0.001
Male	3049(44.3)	2722(39.2)	2488(36.3)	2439(37.0)	
Female	3828(55.7)	4224(60.8)	4359(63.7)	4151(63.0)	
**Smokers(n,%)**					<0.001
Regularly	719(10.5)	1181(17.0)	1750(25.6)	2810(42.6)	
Occasionally	282(4.1)	325(4.7)	306(4.5)	317(4.8)	
Formerly	2691(39.1)	2519(36.3)	2269(33.1)	1742(26.4)	
Never	3185(46.3)	2921(42.1)	2522(36.8)	1721(26.1)	
**History of diabetes(n,%)**	447(6.5)	309(4.4)	209(3.1)	217(3.3)	<0.001
**History of CHD(n,%)**	113(1.6)	134(1.9)	125(1.8)	145(2.2)	0.036
**Anti-hypertensive medication(n,%)**	1287(18.7)	1253(18.0)	1190(17.4)	1156(17.5)	0.044
**Anti-lipid medication(n,%)**	232(3.4)	208(3.0)	212(3.1)	186(2.8)	0.100
**Anemia(n,%)**	218(3.2)	186(2.7)	185(2.7)	224(3.4)	0.465
**Waist circumference(cm)**	85.7 ± 12.7	84.6 ± 15.8	83.4 ± 12.7	82.7 ± 16.3	<0.001
**Systolic blood pressure(mmHg)**	141.5 ± 19.6	141.1 ± 20	141.1 ± 20.2	141 ± 20.2	0.129
**Diastolic blood pressure(mmHg)**	86.4 ± 9.8	85.6 ± 9.9	85.3 ± 10.0	84.9 ± 10.0	<0.001
**ApoA1(mg/dL)**	149.0(134.0,167.0)	153.0(137.0,172.0)	155.0(139.0,175.0)	159.0(140.0,179.0)	<0.001
**ApoB(mg/dL)**	107.0(91.0,125.0)	106.0(89.0,123.0)	105.0(88.0,123.0)	103.0(87.0,120.0)	<0.001
**ApoB/ApoA ratio**	0.72(0.58,0.87)	0.69(0.55,0.84)	0.67(0.54,0.83)	0.65(0.52,0.79)	<0.001
**Total leukocyte count(10**^**9**^**/L)**	5.8(5.0,6.9)	6.0(5.1,7.1)	6.3(5.3,7.4)	6.5(5.4,7.7)	<0.001
**Neutrophil count (10**^**9**^**/L)**	3.5(2.9,4.3)	3.6(3.0,4.5)	3.8(3.0,4.6)	3.9(3.1,4.9)	<0.001
**Lymphocyte count(10**^**9**^**/L)**	1.8(1.5,2.2)	1.9(1.5,2.2)	1.9(1.5,2.3)	1.9(1.6,2.4)	<0.001
**Neutrophil/ Lymphocyte ratio**	1.94(1.54,2.47)	1.95(1.55,2.48)	2.0(1.6,2.5)	2.0(1.6,2.6)	<0.001
**MCV(fL)**	86.1 ± 3.4	88.2 ± 2.9	90.2 ± 3	93.2 ± 3.6	<0.001
**Hemoglobin(g/dL)**	142.6 ± 12.2	141.7 ± 11.9	141.3 ± 11.7	141.3 ± 12.1	<0.001

Continuous variables were shown as mean (SD) or median (Q25,Q75); Categorical variables were shown as number (%).

Baseline characteristics of subjects who did (n = 491) and did not (n = 26,769) develop AAA during follow-up are shown in Supplementary Table 1. Traditional risk markers for CVDs such as age, waist circumference, systolic blood pressure, diastolic blood pressure, ApoB/ApoA1 ratio, total leukocyte count, neutrophil to lymphocyte ratio (NLR), as well as the proportion of men, smokers, history of CHD, history of anti-hypertensive medication, and history of anti-lipid medication were all higher in subjects who developed AAA compared to those who did not.

### RDW and AAA

**Figure 1:**
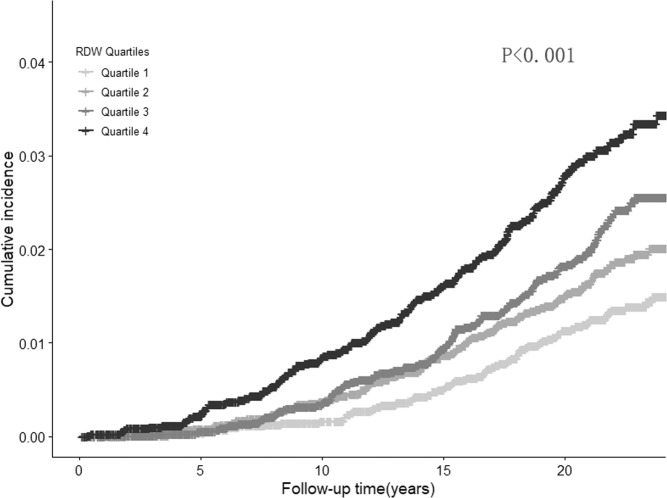
Cumulative incidence of AAA according to quartiles of RDW.

**Figure 2:**
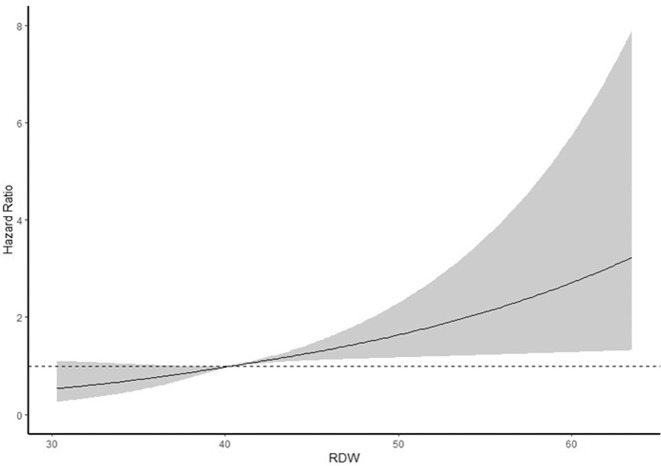
Does-response relationship between RDW and risk of AAA. Adjusted for age, sex, smoking, diabetes, coronary heart disease, waist circumference, systolic blood pressure, ApoB/ApoA1, WBC, hemoglobin, MCV(classified by < 80fl, 80–100fl and >100fl), anti-hypertension medications and lipid-lowing medications.

As shown in Fig. 1, the cumulative incidence rate of AAA was higher in higher quartiles of RDW, and the difference between quartiles increased with time (*p* < 0.001). The association of RDW with the risk of AAA is presented in Table 2. After adjustment for potential confounders in full-adjusted model, individuals in the second, third or highest quartile of RDW, respectively, experienced 23% (HR = 1.23, 95%CI = 0.92,1.64), 40% (HR = 1.40, 95%CI = 1.06,1.86) and 61% (HR = 1.61, 95%CI = 1.20,2.12) higher risk to develop AAA compared to those in the lowest quartile. The analysis modelled by restricted cubic splines showed a clear dose-response relationship between RDW and risk of AAA (Fig. 2). The predictive value of RDW for severe AAA (surgically or ruptured) or non-severe AAA (non-surgically and non-ruptured) was similar. When comparing the highest with lowest quintile, the adjusted HR was 1.58 (95%CI = 1.04,2.41) for severe AAA and 1.60 (95%CI = 1.09,2.35) for non-severe AAA.

**Table 2:** Adjusted hazard ratios for AAA.

RDW	Person years	Incident	Crude HR (95%CI)	Model 1 HR(95%CI)	Model 2 HR(95%CI)	Model 3 HR(95%CI)
**Total AAA**						
Quartile 1	142437	83	Ref	Ref	Ref	Ref
Quartile 2	139988	111	1.38(1.04,1.83)	1.39(1.05,1.85)	1.21(0.91,1.61)	1.23(0.92,1.64)
Quartile 3	134628	131	1.71(1.30,2.26)	1.75(1.33,2.31)	**1.38(1.04,1.83)**	**1.40(1.06,1.86)**
Quartile 4	122294	166	2.46(1.89,3.20)	2.45(1.88,3.20)	**1.58(1.19,2.09)**	**1.61(1.20,2.12)**
P for trend			<0.001	<0.001	0.001	0.001
**Surgically treated or ruptured AAA**						
Quartile 1	142437	37	Ref	Ref	Ref	Ref
Quartile 2	139988	55	1.52(1.00,2.31)	1.58(1.04,2.40)	1.33(0.87,2.02)	1.35(0.89,2.06)
Quartile 3	134628	53	1.53(1.01,2.33)	1.64(1.07,2.51)	1.22(0.79,1.88)	1.24(0.81,1.91)
Quartile 4	122294	76	2.46(1.66,3.65)	2.59(1.74,3.87)	**1.57(1.03,2.39)**	**1.58(1.04,2.41)**
P for trend			<0.001	<0.001	0.062	0.059
**Non-surgically treated and non-ruptured AAA**						
Quartile 1	142437	46	Ref	Ref	Ref	Ref
Quartile 2	139988	56	1.26(0.86,1.87)	1.25(0.84,1.85)	1.11(0.75,1.65)	1.13(0.76,1.67)
Quartile 3	134628	78	1.86(1.29,2.68)	1.84(1.27,2.65)	**1.52(1.04,2.21)**	**1.54(1.06,2.24)**
Quartile 4	122294	90	2.45(1.72,3.49)	2.35(1.64,3.37)	**1.59(1.08,2.32)**	**1.60(1.09,2.35)**
P for trend			<0.001	<0.001	0.006	0.005

Model 1: age, sex adjusted.

Model 2: Model 1 + smoking + diabetes + CHD + waist circumference + systolic blood pressure + ApoB/ApoA1 ratio + WBC + hemoglobin + MCV (classified by <80fl, 80–100fl and >100fl).

Model 3: Model 2 + anti-hypertension medications + lipid-lowing medication.

In the sensitive analysis, we excluded anemic individuals (hemoglobin < 130 g/L in men and <120 g/L in women, respectively)^11^. After multiple adjustments, individuals in the highest quartile still had increased risk of future AAA when compared to the lowest quartile (HR = 1.52, 95%CI = 1.21,2.51), as shown in Supplementary Table 2. Furthermore, the results were essentially unchanged when excluding individuals with very high red blood cell counts (>6.1×10^9^/L in men and >5.4×10^9^/L in women, respectively)^12^ (HR = 1.60, 95%CI = 1.20,2.14; Supplementary Table 3).

We further stratified analysis by smoking status in Supplementary Table 4. The predictive value of RDW for AAA risk was significant in current smokers, but not in former smokers or never smokers. Among current smokers, individuals in the highest quartile of RDW had 1.68 times higher risk of AAA as compared to those in the lowest quartile (95%CI = 1.18,2.38) in the full-adjusted model.

## Discussion

Increased values of RDW have been associated with both increased risk of developing different cardiovascular diseases and poor prognosis in patients with established cardiovascular disease^4,5^. This study demonstrated a positive association between RDW and incidence of AAA. The association was consistent when RDW was modeled as continuous or categorical variables, and the risk of AAA by RDW displayed a dose dependent pattern.

We can only speculate about the reasons for the increased risk of AAA in individuals with high RDW. One possibility is that inflammation could be a common cause of high RDW and AAA. On one hand, high RDW has been associated with systemic inflammation^13^, and inflammation-induced alteration of erythropoiesis could lead to increased anisocytosis, altered life span of the red blood cells (RBCs), and red blood cell membrane deformability^14^. In a study from the NHANES, it was even reported that RDW was a better predictor of CHD mortality than high sensitivity C-reactive protein (hsCRP)^15^. In addition, in a cross-sectional study of patients diagnosed with aneurysms of the ascending aorta, positive correlations were also found between RDW values and systemic inflammatory molecules, including hsCRP, IL-6, TNF- α, MMP-2, and MMP-9 levels^16^. On the other hand, inflammation is suggested to be involved in the pathological processes of AAA^17,18^. These inflammatory factors mentioned above were further related to AAA, especially the MMPs, which could degrade collagen and have been considered as potential key factors in the formation of AAA^19–21^. In the present study, we found that RDW was associated with a high leukocyte count. However, RDW was still significantly related to AAA incidence after adjustments for leukocyte count in our study. This suggests that RDW is associated with other pathophysiological mechanisms, and not only a marker of inflammation^13^.

Oxidative stress in the aortic wall might be another potential pathway linking high RDW and AAA. Oxidative stress could lead to higher level of RDW through increased hemolysis and shortened erythrocyte life-span^22,23^. Besides, oxidative stress is one of the dominant characteristics of AAA, and can provoke both smooth muscle cell death and collagen fiber degradation^18,24^. Animal studies indicate that systemic use of anti-oxidative drugs could prevent the formation and progression of AAA^25^. Oxidative stress could thus be a potential mediator behind the observed association between RDW and AAA, although direct evidence from human or animal studies is lacking, and therefore more investigations are required.

It is also possible that preclinical stages of AAA could be associated with reduced survival of the red blood cells. It is well known that blood clots can occur in aortic aneurysms due to altered flow conditions, and that such thrombi have potential biochemical and biomechanical effects on both blood components and the aortic wall^26,27^. AAA could also be associated with impaired endothelial function and other features of vascular aging^16^, which in turn, could cause mechanical stress to the erythrocytes. Hypothetically, early vascular changes in individuals with high risk of AAA could affect the life span of the red cells and thereby the RDW.

Hematopoietic nutrients deficiency might play a role in the relationship between RDW and AAA as well. As we know, both iron deficiency and vitamin B12/folate deficiency anemia are main causes of elevated RDW^1^. The iron-structured hemoglobin could be released in the aortic wall to promote AAA and cause anemia^28^. Vitamin B12 has been shown to correlate inversely with aneurysm diameter in patients with AAA^29^. Moreover, folate has been shown to reduce the incidence of AAA in animal model^30^. However, our results were essentially unchanged after adjustments for hemoglobin concentrations and in the analysis excluding individuals with anemia. Low hemoglobin and anemia therefore seem to be an unlikely explanation for the relationship between RDW and AAA in this study.

Smoking is associated with high RDW values, and is also a major risk factor for AAA^31–33^. Smoking could induce systemic inflammation and oxidative stress, thus elevating RDW through effects upon RBCs^31,34^. Meanwhile, smoking could increase proteolytic activity, damaging the aortic wall^18,24^. Therefore, smoking status might play a role in the association between RDW and the risk of AAA. After stratification by smoking status, we found that RDW was predictive for AAA in smokers but not significantly in non-smokers. We can therefore conclude that RDW is a stronger risk factor for AAA in smokers than in non-smokers. However, the number of AAA endpoints was limited in non-smokers and we cannot rule out that the non-significant results in non-smokers could be due to limited statistical power.

This is a prospective, population-based study with large sample size and long-term follow-up, allowing us to eliminate causal inversion and recall bias, as well as ensure statistical power. Information of AAA diagnosis was retrieved from register systems with national coverage, which contributes to the accuracy of outcome. There are still some limitations of this study, however. Firstly, misclassification of outcome could not be completely excluded as some aneurysm-carriers might have not been diagnosed due to the symptomless characteristics of AAA in early stages of the disease. Secondly, residual confounding bias could not be avoided, as levels of potential confounders like serum folate, vitamin B12, and iron were not available in our dataset.

## Conclusion

In conclusion, elevated RDW was significantly associated with an increased risk of developing AAA in a general population, with a dose-response relationship. The predictive effect of AAA was even stronger in current smokers. However, the underlying pathophysiological mechanisms connecting RDW and AAA remain largely unknown.

## Methods

### Study population

The Malmö Diet and Cancer study (MDCS) is a prospective cohort study, recruiting participants from the general population in Malmö, a city in south of Sweden. A total of 30,447 individuals attended the baseline examinations between March 1991 and September 1996, including peripheral venous blood samples, physical examination, and a self-administered questionnaire. For the present study, complete information on covariates and outcome was available for 27,308 participants. Twenty-four subjects were excluded to rule out extreme blood indices or laboratory errors. We further excluded 24 subjects with history of AAA prior to the baseline examination. The final study population consisted of 27,260 participants (10,698 males and 16,562 females, aged 45–73 years) **(**Supplementary Fig. 1**)**.

All participants gave informed written consent. The study was approved by the ethics committee at Lund University (LU 51/90, 166/2007) and in accordance with the 1964 Helsinki declaration and its later amendments or comparable ethical standard.

### Baseline examination

At the physical examination, waist circumference was measured with standard methods. Blood pressure was measured after 10 minutes of rest in the supine position. Information about medical history and current medication was assessed in a self-administered questionnaire. Smoking status was categorized into current (including daily or occasional) smoker, former smoker, and never smoker. History of CHD was obtained from the diagnosis linked to register systems. Diabetes mellitus (DM) was defined as a self-reported physician’s diagnosis, or self-reported use of antidiabetic medications, or a diagnosis of diabetes from national or regional hospital registers.

### Laboratory tests

Venous blood samples were drawn from the cubital vein. Hemoglobin, white blood cell (WBC) and its subtypes count, and erythrocyte diameter were analyzed in fresh, heparinized blood, using a fully automated assay (SYSMEX K1000 hematology analyzer; TOA Medical Electronics, Kobe, Japan). RDW was calculated as the width (fL) of the erythrocyte distribution curve at a relative height of 20% above the baseline^35^. Apolipoproteins A1 (ApoA1) and B (ApoB) were measured at Quest Diagnostics (San Juan Capistrano, CA). ApoB/ApoA1 ratio was calculated in the same blood sample.

### Ascertainment of AAA

Follow-up for participants began at the date of baseline examination, and ended at the date of death, migration from Sweden, end of follow-up (December 31st, 2016), or diagnosis of AAA, whichever came first. We obtained the information about diagnosis of AAA by linking to Swedish national registers (the Swedish Inpatient Register, the hospital-based outpatient register and the Cause of Death Register). All datasets were linked using the 10-digit personal identification number which is unique to each Swedish resident. The diagnoses are coded using a Swedish revision of the International Classification of Disease (ICD). The ninth edition (ICD-9) was used between 1987 and 1996, and the 10th edition (ICD-10) has been used since 1997. Incident of AAA was defined based on ICD-9 codes 441D-441E and ICD-10 code I713-I716 or death attributable to AAA. Moreover, AAA was classified as severe (surgically repaired or ruptured) and non-severe (not surgically repaired, not ruptured) subtypes. Information about patients who underwent a surgical procedure for AAA was also obtained from the registers.

A validation of the diagnoses or surgical procedures was performed by review of records for 98 patients with a diagnosis of AAA from Skåne University hospital in Malmö between 1st Jan and 31st December 2016, as summarized in Supplementary Table 5 (modified from Bergwall *et al*.)^36^.

### Statistical analyses

The study population was divided into quartiles based on baseline RDW values (Quartile 1: 28.1–38.4 fL, Quartile 2: 38.5–40.4 fL, Quartile 3: 40.5–42.7 fL, and Quartile 4: 42.8–72.9 fL). Cross-sectional relations of RDW quartiles to baseline characteristics were analyzed using general linear model for continuous variables and logistic regression for binary variables. P-values from trend tests across quartiles were used. Tests for baseline differences of potential confounders between those without and with incident AAA were made using 2-sample t tests (continuous variables) or the chi-square test (binary variables). Cox proportional hazard regression models were used to estimate hazard ratios (HR) with 95% confidence intervals (CI) for AAA across quartile of RDW, and lowest RDW quartile was used as reference group. Subgroup analysis was performed for severe AAA (surgically or ruptured) or non-severe AAA (non-surgically and non-ruptured). Additionally, a plot visualizing the cumulative incidence of AAA according to quartiles of RDW was made. Moreover, dose-response relationship was visualized by using restricted cubic splines, which is a method to test the hypothesis that the relationship is not linear. In this plot, RDW was modelled with a three-degrees of freedom smoothing spline fit in a Cox proportional hazard model using R. And stratified analysis was further conducted by smoking status.

Since anemia and dysregulation of erythropoiesis potentially could affect RDW, we performed a sensitivity analysis after exclusion of individuals with anemia (hemoglobin < 130 g/L in men and <120 g/L in women) and another sensitivity analysis after excluding individuals with erythrocytosis (erythrocyte counts >6.1×10^9^/L in men and >5.4×10^9^/L in women).

All analyses were performed using SPSS version 22.0, and figures were plotted in R.

## Supplementary information

Supplementary Information.
