# Supplementary material for: Red Cell Distribution Width is Associated with Future Incidence of Abdominal Aortic Aneurysm in a Population-Based Cohort Study
Source: Sci Rep. 2020 Apr 29;10:7230. doi: 10.1038/s41598-020-64331-7 (PMC7190826; doi:10.1038/s41598-020-64331-7)
Supplement: Supplementary file 1 — Supplementary Information. [file 41598_2020_64331_MOESM1_ESM.docx]

**Red Cell Distribution Width is Associated with Future Incidence of Abdominal Aortic Aneurysm in a Population-Based Cohort Study**

Jun Xiao, Yan Borné, Anders Gottsäter, Jingxue Pan, Stefan Acosta, Gunnar Engström

| **Supplementary Table 1. Baseline characteristics stratified according to future development of AAA** | | | |
| --- | --- | --- | --- |
| **AAA** | **No AAA (n=26769)** | **Incident AAA (n=491)** | **P** |
| **Age(years)** | 58.1±7.6 | 61.0±6.7 | <0.001 |
| **Sex(n,%)** |  |  | <0.001 |
| Male | 10327(38.6) | 371(75.6) |  |
| Female | 16442(61.4) | 120(24.4) |  |
| **Smoking(n,%)** |  |  | <0.001 |
| Regularly | 6205(23.2) | 255(51.9) |  |
| Occasionally | 1202(4.5) | 28(5.7) |  |
| Formerly | 9062(33.9) | 159(32.4) |  |
| Never | 10300(38.5) | 49(10.0) |  |
| **History of diabetes(n,%)** | 1161(4.3) | 21(4.3) | 0.987 |
| **History of CHD(n,%)** | 474(1.8) | 43(8.8) | <0.001 |
| **Anti-hypertensive medication(n,%)** | 4737(17.7) | 149(30.3) | <0.001 |
| **Anti-lipid medication(n,%)** | 785(2.9) | 53(10.8) | <0.001 |
| **Anemia(n,%)** | 804(3.0) | 9(1.8) | 0.141 |
| **Waist circumference(cm)** | 84.0±14.5 | 91.9±12.4 | <0.001 |
| **Systolic blood pressure(mmHg)** | 141.1±20.0 | 147.8±20.0 | <0.001 |
| **Diastolic blood pressure(mmHg)** | 85.5±9.9 | 92.2±9.8 | <0.001 |
| **ApoA1(mg/dL)** | 154.0(138.0,174.0) | 138.0(124.0,155.0) | <0.001 |
| **ApoB(mg/dL)** | 105.0(89.0,123.0) | 118.0(103.0,134.0) | <0.001 |
| **ApoB/ApoA1 ratio** | 0.68(0.54,0.83) | 0.85(0.72,1.01) | <0.001 |
| **Total leukocyte count(10^9^/L)** | 6.1(5.2,7.3) | 7.0(5.8,8.3) | <0.001 |
| **Neutrophil count (10^9^/L)** | 3.7(3.0,4.6) | 4.2(3.4,5.3) | <0.001 |
| **Lymphocyte count(10^9^/L)** | 1.9(1.5,2.3) | 2.0(1.6,2.4) | <0.001 |
| **NLR** | 1.96(1.56,2.52) | 2.09(1.67,2.69) | <0.001 |
| **MCV(fL)** | 89.4±4.1 | 90.5±4.5 | <0.001 |
| **Hemoglobin(g/dL)** | 141.6±12.0 | 149.4±11.7 | <0.001 |
| **RDW** | 40.7±3.4 | 41.7±3.5 | <0.001 |

Continuous variables were shown as mean (SD) or median (Q25,Q75); Categorical variables were shown as number (%). Natural logarithm transformation was applied for variables with non-normal distribution. Analysis of t-test was used for continuous variables and Chi-square test used for categorical variables.

| **Supplementary Table 2. Adjusted hazard ratios for AAA excluding the anemia individuals (n=26447)** | | | | | | |
| --- | --- | --- | --- | --- | --- | --- |
| **RDW** | **Person years** | **Incident** | **Crude HR (95%CI)** | **Model 1 HR(95%CI)** | **Model 2 HR(95%CI)** | **Model 3 HR(95%CI)** |
| **Total AAA** |  |  |  |  |  |  |
| Quartile 1 | 137916 | 80 | Ref | Ref | Ref | Ref |
| Quartile 2 | 136401 | 110 | 1.41(1.06,1.88) | 1.43(1.07,1.92) | 1.24(0.93,1.66) | 1.26(0.94,1.69) |
| Quartile 3 | 131123 | 130 | 1.75(1.33,2.32) | 1.81(1.37,2.40) | **1.43(1.07,1.90)** | **1.45(1.09,1.93)** |
| Quartile 4 | 118499 | 162 | 2.48(1.90,3.25) | 2.51(1.91,3.30) | **1.60(1.20,2.13)** | **1.62(1.21,2.15)** |
| P for trend |  |  | <0.001 | <0.001 | 0.001 | 0.001 |

Model 1: age, sex adjusted

Model 2: Model 1 + smoking + diabetes + CHD +waist circumference + systolic blood pressure + ApoB/ApoA1 ratio + WBC + hemoglobin + MCV (classified by <80fl, 80-100fl and >100fl)

Model 3: Model 2 + anti-hypertension medications + lipid-lowing medication

| **Supplementary Table 3. Adjusted hazard ratios for AAA excluding the erythrocytosis individuals (n=27092)** | | | | | | |
| --- | --- | --- | --- | --- | --- | --- |
| **RDW** | **Person years** | **Incident** | **Crude HR (95%CI)** | **Model 1 HR(95%CI)** | **Model 2 HR(95%CI)** | **Model 3 HR(95%CI)** |
| **Total AAA** |  |  |  |  |  |  |
| Quartile 1 | 140966 | 81 | Ref | Ref | Ref | Ref |
| Quartile 2 | 139167 | 109 | 1.38(1.04,1.84) | 1.40(1.05,1.87) | 1.22(0.91,1.63) | 1.24(0.92,1.65) |
| Quartile 3 | 134209 | 131 | 1.74(1.32,2.30) | 1.79(1.35,2.37) | **1.41(1.06,1.88)** | **1.43(1.08,1.90)** |
| Quartile 4 | 122057 | 164 | 2.47(1.89,3.22) | 2.47(1.89,3.24) | **1.59(1.20,2.12)** | **1.60(1.20,2.14)** |
| P for trend |  |  | <0.001 | <0.001 | 0.001 | 0.001 |

Model 1: age, sex adjusted

Model 2: Model 1 + smoking + diabetes + CHD +waist circumference + systolic blood pressure + ApoB/ApoA1 ratio + WBC + hemoglobin + MCV (classified by <80fl, 80-100fl and >100fl)

Model 3: Model 2 + anti-hypertension medications + lipid-lowing medication

| **Supplementary Table 4. Adjusted hazard ratios for AAA stratified by smoking status** | | | | | | |
| --- | --- | --- | --- | --- | --- | --- |
| **RDW** | **Person years** | **Incident** | **Crude HR (95%CI)** | **Model 1 HR(95%CI)** | **Model 2 HR(95%CI)** | **Model 3 HR(95%CI)** |
| **Smoking status** |  |  |  |  |  |  |
| **Current smokers** |  |  |  |  |  |  |
| Quartile 1 | 38812 | 62 | Ref | Ref | Ref | Ref |
| Quartile 2 | 36940 | 62 | 1.07(0.75,1.52) | 1.07(0.76,1.53) | 1.05(0.73,1.50) | 1.05(0.73,1.50) |
| Quartile 3 | 36141 | 81 | 1.44(1.03,2.00) | 1.47(1.05,2.04) | **1.60(1.14,2.24)** | **1.56(1.12,2.19)** |
| Quartile 4 | 32777 | 78 | 1.59(1.14,2.22) | 1.49(1.06,2.09) | **1.71(1.21,2.43)** | **1.68(1.18,2.38)** |
| P for trend |  |  | 0.002 | 0.006 | <0.001 | <0.001 |
| **Former smokers** |  |  |  |  |  |  |
| Quartile 1 | 49881 | 43 | Ref | Ref | Ref | Ref |
| Quartile 2 | 44388 | 32 | 0.84(0.53,1.33) | 0.80(0.50,1.26) | 0.86(0.54,1.37) | 0.89(0.56,1.42) |
| Quartile 3 | 44596 | 43 | 1.14(0.75,1.74) | 1.06(0.69,1.62) | 1.24(0.80,1.91) | 1.31(0.85,2.03) |
| Quartile 4 | 43230 | 41 | 1.14(0.74,1.75) | 0.95(0.61,1.47) | 1.08(0.68,1.70) | 1.13(0.72,1.79) |
| P for trend |  |  | 0.343 | 0.905 | 0.437 | 0.319 |
| **Never smoking** |  |  |  |  |  |  |
| Quartile 1 | 55362 | 9 | Ref | Ref | Ref | Ref |
| Quartile 2 | 54160 | 15 | 1.72(0.76,3.94) | 1.67(0.73,3.83) | 1.81(0.79,4.17) | 1.81(0.79,4.17) |
| Quartile 3 | 52178 | 11 | 1.34(0.55,3.23) | 1.20(0.49,2.94) | 1.32(0.54,3.23) | 1.32(0.54,3.23) |
| Quartile 4 | 50882 | 14 | 1.79(0.77,4.13) | 1.40(0.59,3.32) | 1.80(0.75,4.30) | 1.77(0.74,4.22) |
| P for trend |  |  | 0.285 | 0.679 | 0.329 | 0.345 |

Model 1: age, sex adjusted

Model 2: Model 1 + diabetes + CHD +waist circumference + systolic blood pressure + ApoB/ApoA1 ratio + WBC + hemoglobin + MCV (classified by <80fl, 80-100fl and >100fl)

Model 3: Model 2 + anti-hypertension medications + lipid-lowing medications

| **Supplementary Table 5.** Validation of patients with AAA and ruptured AAA (rAAA) | | | |
| --- | --- | --- | --- |
| **Characteristics** | **AAA** (n=80) | **rAAA** (n=18) | *p* value |
| **Confirmed diagnosis, (%)** | 76/80 (95) | 17/18 (94.4) | 1.000 |
| **Median (IQR) age; years** | 76 (71 – 80) | 84 (78 – 85) | <0.001 |
| **Male sex, (%)** | 58/76 (76.3) | 12/17 (70.6) | 0.620 |
| **Operated at the time of diagnosis, (%)** | 5/76 (6.6) | 10/17 (58.8) | <0.001 |
| **Initial mode of diagnosis, (%)** |  |  |  |
| Imaging due to AAA-related symptoms | 2 | 16 |  |
| Accidental finding at any imaging | 56 | 0 |  |
| Palpation | 12 | 0 |  |
| Autopsy | 0 | 1 |  |
| Organized ultrasound screening for AAA | 2 | 0 |  |
| Non-organized ultrasound screening for AAA | 4 | 0 |  |
| rAAA; ruptured abdominal aortic aneurysm, IQR; interquartile range | | | |

MDCS cohort n=30 447

Baseline AAA n=24

Missing covariables n=3139

(RDW n=2098

Hemoglobin n=1

Smoking status n=322

Blood pressure n=46

ApoB/ApoA1 ratio n=623

Total and differential leukocyte counts n=4

Waist circumference n=42

Follow-up information n=3)

Total leukocyte count >20×10^9^/L n=21

RDW <10 or >100 fL n=2

Hemoglobin < 30 g/L n=1

n=27 260, aged 45-73 years, 39.2% men

**Supplementary Figure 1.** Individuals included in this research
